# High-Quality Samples for Next-Generation Sequencing and PD-L1 Assessment in Non-Small Cell Lung Cancer: The Role of Endobronchial Ultrasound-Guided Transbronchial Needle Aspiration

**DOI:** 10.3390/diagnostics15091064

**Published:** 2025-04-22

**Authors:** Marta Rodríguez González, Juan Carlos Montero, José María Sayagués, Tamara Clavero Sánchez, Jonnathan Roldán Ruiz, Miguel Iglesias Heras, María Belén Rivas Marcos, Mar Abad, Rosa Cordovilla Pérez

**Affiliations:** 1 Department of Pathology, Biomedical Research Institute of Salamanca (IBSAL), University Hospital of Salamanca, 37007 Salamanca, Spain; jcmontero@saludcastillayleon.es (J.C.M.); ppmari@usal.es (J.M.S.); 2 Centro de Investigación Biomédica en Red Cáncer (CIBERONC), 28029 Madrid, Spain; 3 Department of Pulmonology, University Hospital of Salamanca, 37007 Salamanca, Spain; 4 Department of Clinical Oncology, University Hospital of Salamanca, 37007 Salamanca, Spain

**Keywords:** non-small cell lung cancer, endobronchial ultrasound-guided transbronchial needle aspiration, PD-L1, next-generation sequencing, cytological sample, quality

## Abstract

**Background/Objectives**: Recent advances in the treatment of non-small cell lung cancer (NSCLC) have shifted from conventional chemotherapy to targeted therapies aimed at specific genetic mutations, particularly in the adenocarcinoma subtype. These therapies have improved overall survival and quality of life. However, some patients still face barriers to accessing these treatments due to challenges in diagnosing advanced-stage NSCLC. Limited tumor cellularity in small biopsies and cytological samples hinders the ability to perform further molecular analyses. Additionally, the increasing number of genetic alterations requiring testing complicates the diagnostic process. To overcome this challenge, we propose combining endobronchial ultrasound-guided transbronchial needle aspiration (EBUS-TBNA) with next-generation sequencing (NGS) and immunohistochemistry for PD-L1. **Methods**: A total of 120 EBUS-TBNA samples were consecutively collected during the first year of integrating NGS at a reference hospital in Castilla y León, Spain. Depending on the histology and patient characteristics, a total of 67 NGS analyses and 116 PD-L1 determinations were performed. **Results**: The cytological sample obtained in these cases successfully achieved the triple objective proposed by the NCCN for lung cancer (diagnosis, staging, and molecular analysis in a single procedure) in 97% of instances. **Conclusions**: Our study highlights the effectiveness of EBUS-TBNA as a comprehensive, cost-effective, and safe diagnostic tool for NSCLC, successfully achieving the triple objective of diagnosis, staging, and molecular analysis in 97% of cases. The procedure consistently provided high-quality samples for NGS and PD-L1 testing, with minimal complications, reinforcing its value as a reliable approach for optimizing personalized treatment strategies.

## 1. Introduction

The treatment of non-small cell lung cancer (NSCLC) has undergone a true revolution in recent years. From a limited therapeutic arsenal based on indiscriminate chemotherapy, it has evolved into the paradigm of targeted therapy addressing specific genetic alterations in the tumor, particularly in the adenocarcinoma subtype. It has been observed that both overall survival [1] and quality of life [2] improve with these treatments. However, there are still patients who cannot access these therapies. This limitation is partly due to diagnostic challenges in advanced-stage patients, where small biopsies or cytological samples often provide limited tumor cellularity, insufficient for additional studies beyond the primary diagnosis. Additionally, the increasing number of molecular alterations requiring testing exacerbates this issue. This situation may potentially be partially reversed soon with the implementation of lung cancer prevention and early detection or screening programs [3].

Two major advances have addressed these challenges. First, techniques for sample acquisition have been refined through endobronchial ultrasound-guided transbronchial needle aspiration (EBUS-TBNA), now the standard diagnostic and staging method for NSCLC. EBUS-TBNA has replaced mediastinoscopy [4] and transthoracic biopsy due to minimal complications [5,6,7], but not CT-guided core biopsy, which remains the preferred choice for peripheral and/or subpleural lesions [8]. Moreover, EBUS-TBNA enables both diagnosis and staging in a single procedure, saving crucial time. The development of more flexible needles and advanced navigation systems (e.g., radial EBUS, electromagnetic navigation) has improved sample quality and the accessibility of smaller, more peripheral lesions that can be targeted with greater precision [9,10]. Second, the introduction of high-throughput techniques, such as next-generation sequencing (NGS), into pathology laboratories has transformed the analysis of numerous therapeutic targets, maximizing the utility of obtained samples. The discovery of activating EGFR mutations enabled the development of targeted therapies against them [11], paving the way for the design of similar drugs targeting ALK [1], ROS1 [12], and RET [13] rearrangements, as well as KRAS (G12C) [14] and BRAF [15,16] mutations. The standardization of immunotherapy as a treatment for patients without known molecular targets, based on the immunohistochemical expression of PD-L1 [17,18] in tumor cells, has significantly contributed to this field, not only in adenocarcinoma but also in other NSCLC subtypes. NGS allows for the simultaneous identification of multiple alterations in a single test, conserving sample material and reducing laboratory time compared to single-gene approaches. EBUS-TBNA has demonstrated diagnostic success rates of 89–98% in NSCLC patients. While it ensures accurate diagnosis and enables monogenic studies in many cases, limited studies have explored its utility for NGS [19]. This highlights the need to achieve the triple aim of EBUS-TBNA in lung cancer: diagnosis, staging, and molecular analysis in a single procedure, as recommended by the NCCN guidelines in 2018 [20].

Nevertheless, despite advancements in improving molecular diagnostics and the availability of targeted therapies, there is currently no robust scientific evidence defining the minimum criteria for successful sample acquisition. While expert recommendations exist [21], decisions about the location of sample collection, the number of EBUS-TBNA passes required to obtain enough material, the adequate cellularity threshold, or the panels to be tested are left to the discretion of interventional pulmonologists and pathologists. Parallel to the development of targeted drugs, a multitude of tools have been implemented, differing in the type of material analyzed (DNA and/or RNA), enrichment methods (hybrid capture or amplicons), required sample quantity (e.g., 10 ng, 20 ng), or turnaround time, complicating the management of collected samples.

This study performed a retrospective analysis to evaluate the role of EBUS-TBNA in molecular and PD-L1 diagnostics. The focus was on optimal acquisition and handling practices based on the experience of a reference hospital in Castilla y León, Spain, during the first year after the implementation of NGS.

## 2. Materials and Methods

### 2.1. Patients

**Figure 1:**
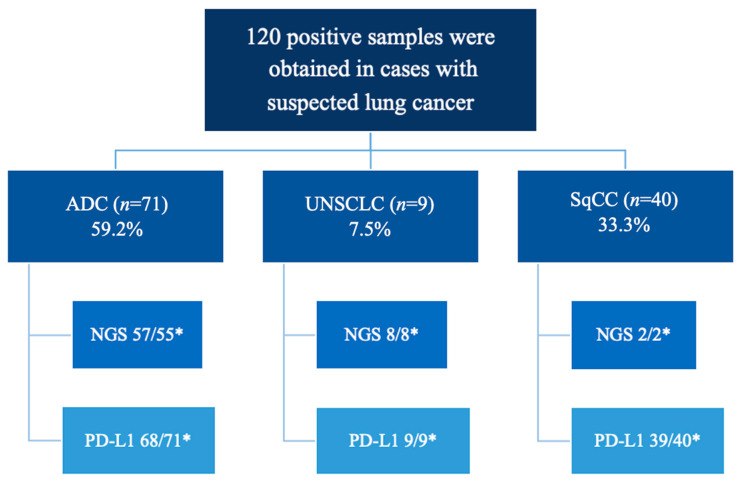
Flowchart of Patients Included in the Study. NSCLC, non-small cell carcinoma; ADC, adenocarcinoma; UNSCLC, undifferentiated non-small cell lung carcinoma; SqCC, squamous cell carcinoma. NGS, next generation sequencing. * requested/performed.

A total of 120 consecutive patients with suspected NSCLC were studied from 1 November 2022 to 31 December 2023 at the Salamanca University Hospital Complex (Figure 1). All patients were over 18 years old and had a clinical and radiological (TC and/or PET-TC) suspicion of advanced lung cancer according to clinical practice guidelines [22,23,24]. They underwent EBUS-TBNA for both diagnostic and staging purposes in a single procedure after obtaining informed consent. Only cases with a histological diagnosis of NSCLC were included for molecular and PD-L1 analysis.

The bronchoscopy procedures were carried out in an Interventional Pulmonology unit using a flexible convex probe bronchoscope (Olympus BF-UC 180F and 190F; Olympus Corp., Tokyo, Japan) under conscious sedation. Specialized 22G or 21G EBUS-TBNA needles (Olympus ViziShot, NA-U401SX-4022, and NA-201SX-4022, Olympus Corp., Tokyo, Japan) were used. Experienced bronchoscopists conducted a comprehensive airway and lymph node exploration for each procedure. Rapid on-site evaluation (ROSE) was performed in 14 patients. All patients underwent a computed tomography (CT) or positron emission tomography-CT (PET-CT) scan before the invasive procedure, as indicated.

At least one cytology block per lesion and/or lymph node station was obtained for each case and fixed in 10% buffered formalin for a minimum of 6 h for procedures performed from Monday to Thursday and for a maximum of 72 h for specimens collected on Fridays. The best sample, as determined by an expert pathologist, was selected for immunohistochemical and molecular analyses. The tumor cell percentage was calculated as a proportion of the total nucleated cellularity in the sample by two expert pathologists.

For each case, a hematoxylin and eosin (H&E) slide was prepared, and the minimal immunohistochemical staining needed to confirm the suspected diagnosis was performed. This included TTF1 (Leica Microsystems Ltd., Milton Keynes, UK, diluted) and/or p40 (A. Menarini Diagnostics, San Diego, CA, USA, 1:200 dilution) using the automated Bond Polymer Refine Detection system (Leica Microsystems (UK) Ltd., Milton Keynes, UK), following the manufacturer’s instructions. Diagnoses were made according to the 5th Edition of the WHO Classification of Thoracic Tumors [25] and staging followed the 8th Edition of the IASLC guidelines [26].

Clinical characteristics (sex, age, smoking history, radiological staging) and bronchoscopy details (explored stations, lymph node and lesion features, number of passes, equipment used, needle size, and the presence of a pathologist in the room) were collected for all patients. Additionally, pathological data (diagnosis, immunohistochemistry, tumor cell percentage, sample quality metrics, NGS, and PD-L1 results) were recorded.

Informed written consent was obtained from the patient to gain access to their data, in accordance with the Declaration of Helsinki. This study was approved by the local Ethics Committee at the University Hospital of Salamanca (Salamanca, Spain; code PI 2023 07 1385; year, 2023). The anonymized database has been uploaded to Zenodo and can be accessed using the following identification number: 10.5281/zenodo.14983303.

### 2.2. Genetic Testing and PD-L1 Analysis

For patients with a diagnosis of NSCLC and a minimum of 50 to 100 tumor cells, PD-L1 immunohistochemical analysis was performed using the 22C3 clone from Dako on the DAKO platform (Dako, Carpenteria, CA, USA). Results are expressed as the percentage of tumor cells showing complete or incomplete membrane staining of any intensity.

### 2.3. Next-Generation Sequencing Study

Following consensus guidelines SEAP-SEOM [18] (Spanish Society of Pathology and Spanish Society of Medical Oncology) and criteria set by the Molecular Committee for Solid Tumors (MCST) at Salamanca University Hospital, NGS analysis was performed on cases with non-squamous NSCLC histology or squamous histology in non-smokers and/or patients younger than 50 years. Eligible samples required a tumor cellularity of over 30% compared to nucleated cellularity, with minimal necrosis.

#### 2.3.1. Sample Preparation and Nucleic Acid Extraction

Between 1 and 6 sections of 5 µm thick FFPE samples were used for DNA and RNA extraction. Sections were deparaffinized in 1 mL xylene at 50 °C, washed with 100% ethanol, and dried at 55 °C. Tissue lysis was performed overnight at 55 °C using a proteinase K solution, followed by 1 h at 90 °C. Subsequently, 200 µL of the lysate was transferred to a plate for automated nucleic acid extraction using the Genexus System (Thermo Fisher Scientific; Waltham, MA, USA). DNA and RNA concentrations (10 ng, measured with Qubit) were assessed, and only samples with concentrations above 0.67 ng/µL were sequenced.

#### 2.3.2. Sequencing and Analysis

Then, the mixture containing nucleic acids and sequencing reagents was loaded into the Ion Torrent™ Genexus™ Integrated Sequencer (Thermo Fisher Scientific; Waltham, MA, USA). The Oncomine Precision Assay (OPA) panel was used to detect hotspot mutations, copy number variations (CNVs), and gene fusions across 50 cancer-related genes using the Ion Torrent GX5 chip. Data were analyzed automatically with the Ion Torrent Genexus software 6.8.0. and the Oncomine™ Reporter software 2024.01.006, and results were filtered using the Variant Matrix Summary 5.16. This summary included mutations, amino acid changes, allele frequencies, fusion reads, and CNVs. Only results meeting recommended quality parameters for FFPE samples were included (Table S1).

### 2.4. Statistical Analysis

For statistical analysis, SPSS software version 21 (IBM Corp., Armonk, NY, USA) was used. For continuous variables with a normal distribution, Student’s *t* test was applied, while for those that did not follow a normal distribution, the Mann–Whitney U test was used. *p*-value < 0.05 (or p corrected by Pearson, as appropriate) was considered statistically significant.

## 3. Results

Out of the 120 patients who underwent EBUS-TBNA from November 2022 to December 2023, all were diagnosed with non-small cell lung cancer (NSCLC) (Figure 1). The clinical and biological characteristics of the cohort are summarized in Table 1. A pathologist was present in the room in only 14 cases (11.7%). The average number of passes per explored region or lesion was three (range two to four, or three in all cases). The equipment used in most cases was the Olympus BF-UC 190F (64 cases, 62.2%), and a 21G needle was used in 115 cases (96.6%).

The main complications observed in patients during the procedure were minor and related to sedation, such as mild oxygen desaturation or non-severe respiratory depression. These were easily corrected, and it was not necessary to interrupt the test. There were no cases of pneumomediastinum, mediastinitis, or infection, nor were there any instances of significant hemorrhage or bleeding.

In our institution, the average turnaround time for the pathology report is 3 days (ranging from 2 to 5 days). Pulmonologists indicate the patient’s stage in the request accompanying the samples, so if metastatic disease is identified, an automatic request for PD-L1 and/or NGS testing is generated. The report for these tests is issued within 5 to 7 days, with exceptions in cases where additional techniques are required to confirm specific alterations. Therefore, the total time for a complete diagnosis from the arrival of the EBUS-TBNA sample ranges between 7 and 12 days.

**Figure 2:**
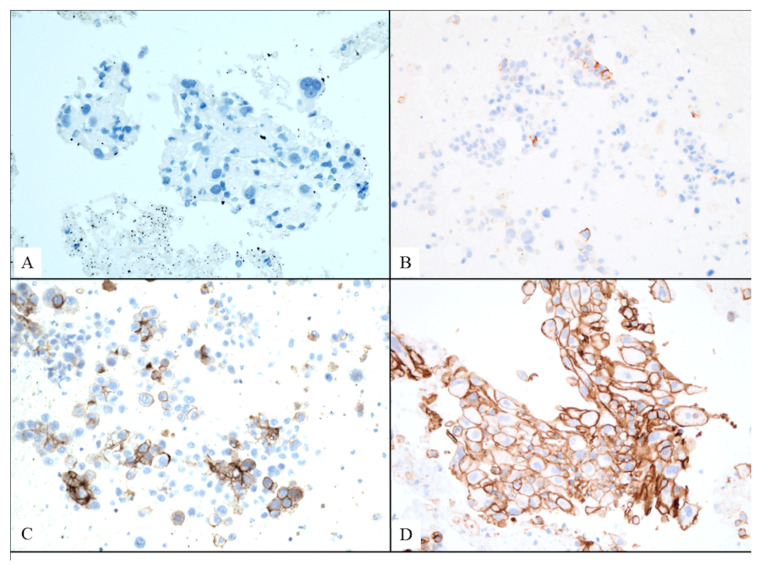
Photographs of immunohistochemistry for PD-L1 (clone 22C3, Dako) performed on cell block samples obtained through EBUS-TBNA sampling with a diagnosis of non-small cell lung carcinoma. (**A**) Completely negative staining. (**B**) Cytoplasmic membrane staining in a few isolated tumor cells, <1%. (**C**) Cytoplasmic membrane staining in <49% of tumor cells (low expressors). (**D**) Cytoplasmic membrane staining in >50% of tumor cells in the sample (high expressors). Micrographs (**A**,**C**,**D**) were acquired at 20× magnification; whereas micrograph (**B**) was adquired at 10x magnification.

The most frequent EBUS-TBNA result was non-squamous carcinoma, with 80 cases (71 adenocarcinomas and 9 undifferentiated non-small cell carcinomas), and 40 cases of squamous cell carcinoma. The squamous carcinoma patients were predominantly male (33 vs. 7; 82.5%) and all were smokers or former smokers. In these patients, the sample was obtained from the pulmonary lesion in 17 cases, with an average size of 30 mm (range 11.4 to 70 mm) measured by EBUS. PET-CT was performed in 12 patients, with an average uptake of 17.4 ± 16.2. Meanwhile, 23 patients had samples taken from metastatic lymph nodes, with an average size of 19.5 ± 10.7 mm. PET-CT was performed in 21 of these 23 patients, with an average standard uptake value (SUV) of 11.6 (range 4.1–23.5). PD-L1 testing was requested for 39 of the 40 squamous histology patients, and the sample was valid in all 39 cases. The result was positive in 25 of them (64.1%), with 8 showing cy-toplasmic membrane positivity of any intensity ≥ 50%, indicating high expression (Figure 2).

According to the consensus guidelines and the CMTS, NGS was requested in only 65 of the 80 non-squamous carcinomas and in two patients with squamous histology. Of the 15 patients with non-squamous histology, NGS was not indicated in 6 patients due to early stage or localized disease. The main reason for not requesting NGS in patients where it was indicated was that it had already been requested on a prior sample (six cases), while in three other cases, clinical reasons led to the preference for monogenic testing specifically for EGFR and ALK. As a result, NGS was requested for a total of 67 patients. All but two of them had a valid sample for NGS (97%) with an average cellularity of 74.9% (range 40–90%). None of the collected variables significantly influenced the success of EBUS-TBNA in obtaining sufficient material for NGS, whereas for PD-L1 (Table 2), the only two parameters that proved significant was the average size of the primary tumor and metastatic lymph node, as measured by EBUS (*p* = 0.000), and the presence of a pathologist in the room (*p* = 0.045).

**Figure 3:**
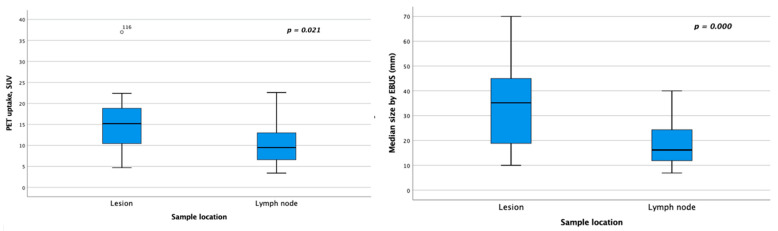
Bar chart showing statistically significant differences between the two sample locations (lesion and lymph node) in the average uptake measured by PET and the average size measured by transbronchial endobronchial ultrasound.

However, when we compared the samples based on their origin (lesion vs. lymph node) (Table 3), we found differences in the stage, mean uptake in the PET-CT, and mean size measured by endoscopic ultrasound regarding the validity of the sample for NGS. In high stages (III and IV), the sample used for NGS was from both the lesion and the lymph node (100% vs. 92%, respectively). In early stages (I and II), the sample was only obtained from the metastatic lymph node (0% from the lesion vs. 8% from the node, with a significance of *p* = 0.036). Furthermore, in samples obtained from the lesion, both the mean lesion uptake (SUV of 14.7 in the lesion vs. 8.9 in the lymph node) and the mean size measured during EBUS-TBNA (26.7 mm in the lesion vs. 13.4 mm in the lymph node) were statistically significant, with *p* < 0.05 (Figure 3). Of the 65 samples with sufficient material to perform the NGS technique, all had adequate quality parameters. These parameters included DNA-related factors such as the number of mapped reads, an average quality score ≥ 20, the mean read length in base pairs, and the uniformity of base coverage, as well as RNA-related factors like the number of mapped reads, the mean read length in base pairs, and the number of RNA expression controls detected (Table 1). When we compared the results of NGS regarding their origin (lesion vs. lymph node), we found that the samples obtained from the lesion had a median RNA read length of 89 (73–92), versus a median of 91 (46–105) for the node. These differences were statistically significant (*p* = 0.038) (Table 3).

Finally, when analyzing the parameters influencing the study of PD-L1 by categorizing them based on the sample site of origin (Table 4), we observed that none of the evaluated parameters significantly affected the validity of the material for performing this tech-nique except for the average size of the lesion compared to the lymph node (26.3 vs. 13.9; *p* = 0.000). Similarly, the results obtained, whether positive or negative, as well as the degree of expression, did not show differences concerning the location of the EBUS-TBNA sampling.

## 4. Discussion

Despite the declining incidence of lung cancer, it remains the leading cause of cancer-related death in both men and women [3,27]. Given the lack of effective population screening and the fact that its symptoms often do not appear until the disease is advanced, most patients are diagnosed at later stages, where diagnostic and curative options are more limited [14,28]. Therefore, it is crucial that these patients, who will not undergo surgical biopsy, are accurately diagnosed from both a histological and molecular perspective, enabling them to receive the best possible treatment and improve their quality of life [29]. The aim of our study was to assess the role of EBUS-TBNA in molecular and PD-L1 diagnostics, focusing on sample acquisition and handling. We reported on the first complete year of using NGS for molecular diagnosis in advanced-stage patients, exclusively utilizing cytology material. The primary limitation of this study is that it was conducted in a single institution. To overcome this, future multicenter studies would be required to validate these findings.

In this context, EBUS-FNA has proven to be an ideal approach, offering an optimal balance between the quality of the material obtained and the associated patient risk. In our study, valid samples were obtained using this method from 97% of patients, in line with previously published series, such as those by Uchimura et al. and by Fernández-Acereño et al., which reported validity rates of 80–90% [19,30,31,32,33]. Moreover, EBUS-TBNA is a technique with minimal complications [7] that can be used in patients at advanced stages with significant functional impairment or high ECOG scores, who are not candidates for surgery and where other techniques would pose higher risks [34,35]. In our series, the complication rate has been low, with minimal desaturation and/or mild respiratory depression related to sedation, consistent with reports in the literature [36]. In addition to these, more severe complications such as mediastinitis, pneumomediastinum, or significant hemorrhage have also been described. However, these are rare and more commonly associated with granulomatous diseases, such as sarcoidosis, making EBUS-TBNA a very safe procedure [37,38,39].

Given the increasing complexity of diagnosing non-small cell lung cancer (NSCLC) at the histological, immunohistochemical, and molecular levels, effective collaboration between pulmonologists and pathologists is crucial for ensuring a streamlined workflow and obtaining high-quality samples, thereby achieving the three primary objectives outlined in the NCCN guidelines in a single procedure [5,20]. In this regard, some authors suggest that the presence of a pathologist in the room, as in the ROSE procedure, plays a crucial role in ensuring the success of molecular analysis of EBUS-TBNA samples [33,40,41,42,43,44]. In our series, only 11.7% samples had pathologist validation, and we did not observe statistically significant differences regarding the ability to perform NGS (*p* = 0.548). This can be partly attributed to the limited number of validations and our well-established sample handling protocol for non-validated specimens, supported by extensive collaborative experience, which eliminates the need for additional passes in patients without ROSE, in contrast to practices described by other authors [33,44,45].

Some articles suggest that the key factor may not be the presence of a pathologist in the room, but rather the number of passes performed [19,43,46]. In this sense, the minimum number of passes per site according to the reviewed literature is three to obtain enough material for the complete histopathological study of the sample [46,47], although some authors, such as Martin-Deleon et al., recommend performing a fourth pass if molecular studies are required [7,30,43,46,47,48]. The average number of passes per station in our series was three, and it did not lead to a significant increase in the cellular percentage or material quality. In our experience, when multiple samples from the same patient are positive, we reserve the one with the highest percentage of tumor cells for NGS and PD-L1, performing immunohistochemical techniques on other regions, provided the cellular morphology is consistent. This ensures that the best sample from the patient is used for molecular and PD-L1 studies.

In our study, the puncture site did not impact the adequacy of material for NGS or PD-L1 analysis. We also found no significant differences between the sample origin (lesion or lymph node) in EBUS-TBNA, except for lesions that had significantly higher average PET-CT uptake and larger sizes on endoscopic ultrasound compared to metastatic lymphadenopathy. This could be explained by the fact that in the lesion, the number of tumor cells and their proportion relative to other cell populations was higher compared to metastatic lymph nodes, where tumor cells typically represent a lower percentage compared to lymphoid cellularity; and it can also be explained by the fact that in small lesions, such as lymph nodes, there is sometimes an underestimation of the SUV, as observed in our series, where the average lesion size measured on PET/CT was significantly larger compared to the average size of metastatic lymph nodes [49,50].

Cytological samples have proven to be highly cost-effective in routine clinical practice. In our series, they enabled the diagnosis of 120 patients and the performance of complementary techniques in 94.2% of them over nearly a year (65 of 67 cases underwent NGS and PD-L1; and in 48 of the remaining 53, PD-L1 testing was performed). Success rates in the literature vary, ranging from 50–60% [7,19] to 80–90% [33,41,46,51,52]. These samples allow easy control of preanalytical variables, ensuring excellent preservation of genetic material [6,32,33], as evidenced by the quality controls we presented.

Regardless of their location, the samples showed a high percentage of alignment with the reference sequence in the reference DNA, as well as an average quality of ≥20, reflecting excellent sequencing base quality. The average read length indicated proper library preparation, without excessive DNA fragmentation. In our series, the uniformity in base coverage of DNA was adequate, allowing accurate detection of variants. The samples obtained through EBUS-TBNA demonstrated solid quality parameters for RNA, which is crucial in lung cancer, given the therapeutic implications of certain fusions like ALK and ROS1. While we did find a minimal difference in the average RNA length between samples obtained from the lesion and those from the lymph node (Table 3), the lesion samples ranged from 73 to 92 base pairs (bp), while the lymph node samples had a significantly higher range (46–105 bp). This could indicate higher sample purity, as metastatic adenopathy samples typically include a proportion of lymphocytes in addition to tumor cells, whereas lesion-derived samples are predominantly composed of tumor cells. These findings underscore the importance of sample origin in interpreting RNA sequencing data, particularly in studies where RNA quality is crucial.

If properly managed, these samples are highly versatile. For example, in pre-digitized extensions, the coverslip can be removed and the sample de-stained for immunohistochemistry or molecular techniques by scraping the surface of the slide [33,53], and centrifuging the needle wash fluid allows the creation of a cell block from the supernatant or pellet [54]. All of this greatly increases the cost-effectiveness of the tumor material obtained, ensuring diagnosis with a reasonable use of immunohistochemistry [25] and optimizing the genetic material extracted for molecular diagnosis [55]. Our findings align with recent studies, including Aljohaney et al., who reported that combining cytology with a cell block improves the diagnostic yield over cytology alone in EBUS-TBNA [56].

NGS offers several advantages over performing monogenic determinations in terms of sample, time, and laboratory resource savings. Depending on the platform, the entire genome or exome can be analyzed, or a more limited panel of specific genes may be used [57,58,59]. Although the former provides a wealth of information, they remain very costly and require a greater amount of genetic material compared to the latter. Furthermore, the information they generate may recommend the use of expensive drugs outside of approved indications [59,60]. From the perspective of public health and how the healthcare system is organized in Spain, particularly in our region, the most cost-effective approach is to use a gene panel that best meets the needs of the laboratory. There are many validated platforms available that standardize results with high analytical sensitivity and can identify a wide range of genomic alterations (single nucleotide variants, insertions and deletions, copy number variations, gene fusions, tumor mutational burden, and microsatellite instability) in a single experiment.

To date, multiple studies support the use of EBUS-TBNA material with valuable samples for molecular techniques and/or PD-L1 testing. However, many of these studies combine different types of samples, such as cell blocks and extensions [6,40,45,51,54]; biopsies, punctures, and aspirates [7,32]; transthoracic cylinders, punctures, and biopsies [5,61]; cryobiopsies [62,63]; and punctures [10]; or even fresh frozen samples obtained by puncture [64]. In this regard, some recent studies have shown that cryobiopsies perform better than EBUS-TBNA; however, they are associated with a higher number of complications [63]. Nevertheless, for other authors, such as Bonatta-Riel et al., when only samples positive for malignancy are analyzed, no statistically significant differences have been observed between EBUS-TBNA and cryobiopsy [62]. Therefore, there is no clear criterion for managing the obtained material, nor for validating these samples, especially concerning the minimum percentage of tumor cellularity required, which often depends on the panel being used.

## 5. Conclusions

In this study, we presented the first complete year of work with NGS for molecular diagnosis in advanced-stage patients using puncture material exclusively at a fourth-level hospital, a regional referral center. We employed a well-established workflow, using a homogeneous patient population regarding sample collection method, material management, NGS and PD-L1 quality standards, and the applied panel. The cytological sample obtained in these cases successfully achieved the triple objective proposed by the NCCN for lung cancer in 97% of cases. In addition to an accurate diagnosis, the material obtained was sufficient to perform NGS techniques with the required quality, as well as immunohistochemistry techniques for PD-L1 without significant complications, regardless of the location; except for RNA, which in our series appears to be better preserved in the lymph nodes, with minimal differences compared to the primary lesion. This demonstrates that EBUS-TBNA is highly cost-effective and safe for the patient.

## Figures and Tables

**Table 1:** Characteristics of patients with EBUS-TBNA positive for non-small cell lung cancer (*n* = 120).

Characteristics of Patients		N (%) or Median (Range)
Age, years		
		70.3 (45–88)
Gender		
	Male	87 (72.5%)
	Female	33 (27.5%)
Smoking history		
	Never smoker	15 (12.5%)
	Former smoker	34 (28.3%)
	Current smoker	71 (59.2%)
Stage (based on 8th ed. of the AJCC) *		
	Ia	1 (0.8%)
	Ib	2 (1.7%)
	IIb	4 (3.3%)
	IIIa	20 (16.7%)
	IIIb	24 (20.0%)
	IV	69 (57.5%)
PET-CT		
	Yes	83 (69.2%)
	No	37 (30.8%)
PET SUVmax values		
	Primary tumor	14.6 (2.9–58.9)
	Lymph node	8.9 (3.4–23.5)
Sample location		
	Primary tumor	39 (32.5%)
	Lymph node	81 (67.5%)
Size by EBUS (mm)		
	Primary tumor	26.7 (10–70)
	Lymph node	13.4 (6.7–43.8)
ROSE		
	Yes	14 (11.7%)
	No	106 (88.3%)
Equipment * (*n* = 119)		
	180	45 (37.5%)
	190	74 (61.7%)
Needle type * (*n* = 119)		
	21G	115 (95.8%)
	22G	4 (3.3%)
Histological tumor type		
	Squamous cell carcinoma	40 (33.3%)
	Adenocarcinoma	71 (59.2%)
	Undifferentiated non-small cell carcinoma	9 (7.5%)
PD-L1 ** (*n* = 112)		
	0%	42 (37.5%)
	1–49%	46 (41.1%)
	≥ 50%	24 (21.4%)
NGS quality parameters *** (*n* = 65)		
	Mapped reads DNA	1,139,697.0 (12,550.0–5,693,196.0)
	Mean AQ 20 read lenght (bp)	90.0 (69.0–98.0)
	Mean read length (bp)	99.0 (60.0–105.0)
	Uniformity base coverage	97.4 (73.6–100.0)
	Mapped reads RNA	185,670.0 (17,515.0–2,075,290.0)
	Mean read length RNA	91.0 (46.0–105.0)
	RNA expression control detected	7.0 (3.0–7.0)
Detection of treatment target by NGS *** (*n* = 65)		
	Yes	28 (44.4%)
	No	35 (55.6%)
Mutation of *TP53* *** (*n* = 65)		
	Yes	23 (35.4%)
	No	42 (64.6%)
Deaths		
	Yes	32 (26.7%)
	No	88 (73.3%)

AJCC indicates American Joint Committee on Cancer. * Data were collected from 119 out of the 120 total patients in the series. ** A total of 112 PD-L1 tests were performed out of the 116 requested. *** A total of 65 NGS were performed out of the 67 requested.

**Table 2:** Influence of Different Variables on the Acquisition of Valid Samples for NGS and PD-L1 in Patients Diagnosed with NSCLC through EBUS-TBNA sampling.

		NGS			PD-L1		
		Candidates for NGS (*n* = 67) (Median and Range or n and %)	Sample Adequacy (*n* = 65) (Median and Range or n and %)	Significance (*p*)	Candidates for PD-L1 (*n* = 116) (Median and Range or n and %)	Sample Adequacy (*n* = 112) (Median and Range or n and %)	Significance (*p*)
Age, years (median, range)							
		71 (45–88)	70 (45–88)	0.605	70 (53–84)	71 (45–88)	0.912
Gender							
	Male	42 (62.7%)	41 (97.6%)	0.706	84 (72.4%)	81 (96.4%)	0.919
	Female	25 (37.3%)	24 (96.0%)		32 (27.6%)	31 (96.9%)	
Smoking history							
	Never smoker	15 (22.4%)	14 (93.3%)		15 (12.9%)	14 (93.3%)	
	Former smoker	16 (23.9%)	16 (100.0%)	0.549	33 (28.4%)	33 (100.0%)	0.346
	Current smoker	36 (53.7%)	35 (97.2%)		68 (58.7%)	65 (95.6)	
Stage (based on 8th ed. of the AJCC)							
	Ia	1 (1.5%)	1 (100.0%)		1 (0.9%)	1 (100.0%)	
	Ib	1 (1.5%)	1 (100.0%)		2 (1.7%)	2 (100.0%)	
	IIb	2 (3.0%)	2 (100.0%)	0.849	4 (3.4%)	3 (100.0%)	0.560
	IIIa	12 (17.9%)	12 (100.0%)		19 (16.4%)	18 (94.7%)	
	IIIb	17 (25.4%)	17 (100.0%)		24 (20.7%)	24 (100.0%)	
	IV	34 (50.7%)	32 (94.1%)		66 (56.9%)	64 (97.0%)	
PET-CT							
	Yes	40 (59.7%)	39 (97.5%)	0.776	79 (65.5%)	76 (96.2%)	0.800
	No	27 (40.3%)	26 (96.3%)		37 (34.5%)	36 (97.1%)	
PET SUVmax values							
	Primary tumor	15.2 (4.7–37.0)	15.2 (4.7–37.0)	0.979	14.6 (2.9–58.9)	14.7 (2.9–58.9)	0.076
	Lymph node	9.3 (3.4–22.6)	4.9 (3.4–22.6)		9.3 (3.4–23.5)	9.5 (3.4–23.5)	
Sample location							
	Primary tumor	17 (25.4%)	17 (100.0%)	0.402	39 (33.6%)	38 (97.4%)	0.743
	Lymph node	50 (74.6%)	48 (96.0%)		77 (66.4%)	74 (96.1%)	
Size by EBUS (mm)							
	Primary tumor	29.9 (10.0–70.0)	29.9 (10.0–70.0)	0.473	26.7 (10.0–70.0)	25.8 (10.0–70.0)	0.000
	Lymph node	13.2 (6.9–40.0)	16.2 (6.9–40.0)		13.7 (6.7–43.8)	14.5 (6.7–43.8)	
ROSE							
	Yes	10 (14.9%)	10 (100.0%)	0.548	14 (12.0%)	12 (85.7%)	0.045
	No	57 (85.1%)	55 (96.5%)		102 (88.0%)	97 (95.1%)	
Equipment (*n* = 119)							
	180	21 (31.3%)	21 (100.0%)	0.332	43 (37.1%)	43 (100.0%)	0.202
	190	46 (68.7%)	44 (95.7%)		73 (62.9%)	69 (94.5%)	
Needle type (*n* = 119)							
	21G	64 (95.5%)	62 (96.9%)	0.756	112 (96.6%)	108 (96.4%)	0.840
	22G	3 (4.5%)	3 (100.0%)		4 (3.4%)	4 (100.0%)	
Histological tumor type							
	Squamous cell carcinoma	2 (3.0%)	2 (100.0%)		39 (33.6%)	39 (100.0%)	
	Adenocarcinoma	57 (85.1%)	55 (96.5%)	0.835	68 (58.6%)	64 (94.1%)	0.492
	Undifferentiated non-small cell carcinoma	8 (11.9%)	8 (100.0%)		9 (7.8%)	9 (100.0%)	

**Table 3:** Influence of different variables on the acquisition of valid samples for NGS and results in patients diagnosed with NSCLC through EBUS-TBNA sampling based on the collection site.

		Collection Site		Total (*n* = 65)	Significance
		Lesion (*n* = 17)	Lymph Node (*n* = 48)		(*p*)
Age, years (median, range)					
		69.0 (53–78)	71.5 (45–88)	70 (45–88)	0.320
Gender					
	Male	11 (65%)	30 (63%)	41	0.871
	Female	6 (35%)	18 (37%)	24	
Smoking history					
	Never smoker	4 (24%)	10 (21%)	14	
	Former smoker	4 (24%)	12 (25%)	16	0.972
	Current smoker	9 (52%)	26 (54%)	35	
Stage (based on 8th ed. of the AJCC)					
	Ia	0 (0%)	1 (2%)	1	
	Ib	0 (0%)	1 (2%)	1	
	IIb	0 (0%)	2 (4%)	2	0.049
	IIIa	4 (24%)	8 (17%)	12	
	IIIb	9 (52%)	8 (17%)	17	
	IV	4 (24%)	28 (58%)	32	
PET-CT					
	Yes	11 (65%)	29 (58%)	40	0.626
	No	6 (35%)	21 (42%)	27	
PET SUVmax values (*n* = 38)					
		14.7 (2.9–58.9)	8.9 (3.4–23.5)		0.021
Size by EBUS (mm)					
		26.7 (10.0–70.0)	13.4 (6.7–43.8)		0.000
ROSE					
	Yes	3 (18%)	7 (15%)	10	0.764
	No	14 (82%)	41 (85%)	55	
Equipment					
	180	3 (18%)	18 (38%)	21	0.133
	190	14 (82%)	30 (63%)	44	
Needle type					
	21G	16 (94%)	46 (96%)	62	0.772
	22G	1 (6%)	2 (4%)	3	
Histological tumor type					
	Squamous cell carcinoma	0 (0%)	2 (4%)	2	
	Adenocarcinoma	14 (82%)	41 (85%)	55	0.534
	Undifferentiated non-small cell carcinoma	3 (18%)	5 (11%)	8	
NGS quality parameters					
	Mapped reads DNA	1,147,680.0 (147,321.0–1,538,582.0)	1,139,602.5 (12,550.0–5,693,196.0)	1,139,697.0 (12,550.0–5,693,196.0)	0.941
	Mean AQ 20 read lenght (bp)	89.0 (69.0–95.0)	90.0 (84.0–98.0)	90.0 (69.0–98.0)	0.863
	Mean Read Length (bp)	98.0 (60.0–102.0)	99.0 (89.0–105.0)	99.0 (60.0–105.0)	0.775
	Uniformity base coverage	96.2 (91.6–98.9)	97.5 (73.6–100.0)	97.4 (73.6–100.0)	0.624
	Mapped reads RNA	185,670.0 (75,667.0–364,685.0)	182,873.5 (17,515.0–2,075,290.0)	185,670.0 (17,515.0–2,075,290.0)	0.941
	Mean read length RNA (bp)	89.0 (73.0–92.0)	91.0 (46.0–105.0)	91.0 (46.0–105.0)	0.038
	RNA Expression control detected	7.0 (7.0–7.0)	7.0 (3.0–7.0)	7.0 (3.0–7.0)	not applicable
Detection of treatment target by NGS					
	Yes	8 (47%)	20 (44%)	28	0.800
	No	9 (53%)	26 (57%)	35	
Mutation of *TP53*					
	Yes	9 (53%)	34 (71%)	42	0.078
	No	8 (47%)	14 (29%)	23	

**Table 4:** Influence of different variables on the acquisition of valid samples for PD-L1 and results in patients diagnosed with NSCLC through EBUS-TBNA sampling based on the collection site.

		Collection Site		Total (*n* = 116)	Significance
		Lesion (*n* = 38)	Lymph Node (*n* = 78)		(*p*)
Age, years (median, range)					
		71 (53–84)	72 (45–88)	71 (45–88)	0.385
Gender					
	Male	30 (79%)	54 (69%)	84	0.272
	Female	8 (21%)	24 (31%)	32	
Smoking history					
	Never smoker	4 (11%)	10 (13%)	14	
	Former smoker	13 (34%)	21 (27%)	34	0.711
	Current smoker	21 (55%)	47 (60%)	68	
Stage (based on 8th ed. of the AJCC)					
	Ia	0 (0%)	1 (1%)	1	
	Ib	0 (0%)	2 (2%)	1	
	IIb	0 (0%)	3 (4%)	2	0.130
	IIIa	6 (16%)	13 (17%)	12	
	IIIb	13 (34%)	11 (14%)	17	
	IV	19 (50%)	48 (62%)	32	
PET-CT					
	Yes	13 (34%)	23 (29%)	36	0.606
	No	25 (66%)	55 (71%)	80	
PET SUVmax values (*n* = 79)					
		14.1 (2.9–58.9)	9.3 (3.4–23.5)	9.9 (2.9–58.9)	0.093
Size by EBUS (mm)					
		26.3 (10.0–70.0)	13.9 (6.7–43.8)	17.2 (6.7–70.0)	0.000
ROSE					
	Yes	3 (8%)	9 (12%)	12	0.545
	No	35 (92%)	69 (88%)	104	
Equipment					
	180	13 (34%)	32 (42%)	21	0.133
	190	25 (66%)	45 (58%)	44	
Needle type					
	21G	37 (97%)	74 (95%)	62	0.448
	22G	1 (3%)	3 (5%)	3	
Histological tumor type					
	Squamous cell carcinoma	17 (45%)	23 (29%)	40	
	Adenocarcinoma	17 (45%)	50 (64%)	67	0.139
	Undifferentiated non-small cell carcinoma	4 (10%)	5 (7%)	9	
Result of immunohistochemistry for PDL1					
	Negative (0, <1%)	11 (29%)	31 (45%)	42	0.180
	Positive ( ≥ 1%)	27 (71%)	43 (55%)	70	
PDL1 expressors (*n* = 70)					
	Low expresión (1–49%)	20 (74%)	26 (60%)	46	0.243
	High expresión ( ≥ 50%)	7 (26%)	17 (40%)	24	

## Data Availability

We are in the process of depositing our database in a publicly accessible repository. The reference number will be provided as soon as it is obtained, during the review process.

## Supplementary Materials

The following supporting information can be downloaded at: https://www.mdpi.com/article/10.3390/diagnostics15091064/s1, Table S1: Recommended metrics for DNA and RNA isolated from tumor FFPE samples.

## Abbreviations

The following abbreviations are used in this manuscript:

NSCLC	Non-small cell lung cancer
EBUS-TBNA	Endobronchial ultrasound-guided transbronchial needle aspiration
NGS	Next-generation sequencing
NCCN	National Comprehensive Cancer Network
CT	Computed tomography
PET-CT	Positron emission tomography-CT
SEAP-SEOM	Spanish Society of Pathology and Spanish Society of Medical Oncology
MCST	Molecular Committee for Solid Tumors
FFPE	Formalin-fixed, paraffin-embedded
DNA	Deoxyribonucleic acid
RNA	Ribonucleic acid
CNV	Copy number variations

## References

[1] Tan A.C., Tan D.S.W. (2022). Targeted Therapies for Lung Cancer Patients with Oncogenic Driver Molecular Alterations. J. Clin. Oncol..

[2] Servetto A., Di Maio M., Salomone F., Napolitano F., Paratore C., Di Costanzo F., Viscardi G., Santaniello A., Formisano L., Bianco R. (2023). Analysis of phase III clinical trials in metastatic NSCLC to assess the correlation between QoL results and survival outcomes. BMC Med..

[3] Bade B.C., Dela Cruz C.S. (2020). Lung Cancer 2020: Epidemiology, Etiology, and Prevention. Clin. Chest Med..

[4] Adams K., Shah P.L., Edmonds L., Lim E. (2009). Test performance of endobronchial ultrasound and transbronchial needle aspiration biopsy for mediastinal staging in patients with lung cancer: Systematic review and meta-analysis. Thorax.

[5] McLean A.E.B., Barnes D.J., Troy L.K. (2018). Diagnosing Lung Cancer: The Complexities of Obtaining a Tissue Diagnosis in the Era of Minimally Invasive and Personalised Medicine. J. Clin. Med..

[6] Bilaçeroğlu S. (2017). Molecular markers in lung cancer: Role of EBUS. Curr. Opin. Pulm. Med..

[7] Diep R., MacDonald M., Cooper R., Grzegorczyk A., Rakocevic R., Chang C.-F., Uy A., Cowgill N., Nieva J.J. (2023). Biopsy Method and Needle Size on Success of Next-Generation Sequencing in NSCLC: A Brief Report. JTO Clin. Res. Rep..

[8] Baratella E., Cernic S., Minelli P., Furlan G., Crimì F., Rocco S., Ruaro B., Cova M.A. (2022). Accuracy of CT-Guided Core-Needle Biopsy in Diagnosis of Thoracic Lesions Suspicious for Primitive Malignancy of the Lung: A Five-Year Retrospective Analysis. Tomography.

[9] Steinfort D.P., Khor Y.H., Manser R.L., Irving L.B. (2011). Radial probe endobronchial ultrasound for the diagnosis of peripheral lung cancer: Systematic review and meta-analysis. Eur. Respir. J..

[10] Tone M., Inomata M., Awano N., Kuse N., Takada K., Minami J., Muto Y., Fujimoto K., Kumasaka T., Izumo T. (2021). Comparison of adequacy between transbronchial lung cryobiopsy samples and endobronchial ultrasound-guided transbronchial needle aspiration samples for next-generation sequencing analysis. Thorac. Cancer.

[11] Rosell R., Moran T., Queralt C., Porta R., Cardenal F., Camps C., Majem M., Lopez-Vivanco G., Isla D., Provencio M. (2009). Screening for Epidermal Growth Factor Receptor Mutations in Lung Cancer. N. Engl. J. Med..

[12] Gendarme S., Bylicki O., Chouaid C., Guisier F. (2022). ROS-1 Fusions in Non-Small-Cell Lung Cancer: Evidence to Date. Curr. Oncol..

[13] Cascetta P., Sforza V., Manzo A., Carillio G., Palumbo G., Esposito G., Montanino A., Costanzo R., Sandomenico C., De Cecio R. (2021). RET Inhibitors in Non-Small-Cell Lung Cancer. Cancers.

[14] Reck M., Carbone D.P., Garassino M., Barlesi F. (2021). Targeting KRAS in non-small-cell lung cancer: Recent progress and new approaches. Ann. Oncol..

[15] Hwang I., Choi Y.-L., Lee H., Hwang S., Lee B., Yang H., Chelakkot C., Han J. (2022). Selection Strategies and Practical Application of BRAF V600E-Mutated Non-Small Cell Lung Carcinoma. Cancer Res. Treat..

[16] Imyanitov E.N., Iyevleva A.G., Levchenko E.V. (2021). Molecular testing and targeted therapy for non-small cell lung cancer: Current status and perspectives. Crit. Rev. Oncol. Hematol..

[17] Patel S.A., Weiss J. (2020). Advances in the Treatment of Non-Small Cell Lung Cancer: Immunotherapy. Clin. Chest Med..

[18] Isla D., Lozano M.D., Paz-Ares L., Salas C., de Castro J., Conde E., Felip E., Gómez-Román J., Garrido P., Enguita A.B. (2022). New update to the guidelines on testing predictive biomarkers in non-small-cell lung cancer: A National Consensus of the Spanish Society of Pathology and the Spanish Society of Medical Oncology. Clin. Transl. Oncol..

[19] Karadzovska-Kotevska M., Brunnström H., Kosieradzki J., Ek L., Estberg C., Staaf J., Barath S., Planck M. (2022). Feasibility of EBUS-TBNA for histopathological and molecular diagnostics of NSCLC—A retrospective single-center experience. PLoS ONE.

[20] Ettinger D.S., Aisner D.L., Wood D.E., Akerley W., Bauman J., Chang J.Y., Chirieac L.R., D’Amico T.A., Dilling T.J., Dobelbower M. (2018). NCCN Guidelines Insights: Non–Small Cell Lung Cancer, Version 5.2018. J. Natl. Compr. Canc. Netw..

[21] Gilbert C.R., Dust C., Argento A.C., Feller-Kopman D., Gonzalez A.V., Herth F., Iaccarino J.M., Illei P., O’Neil K., Pastis N. (2024). Acquisition and Handling of Endobronchial Ultrasound Transbronchial Needle Samples. CHEST.

[22] Rami-Porta R., Call S., Dooms C., Obiols C., Sánchez M., Travis W.D., Vollmer I. (2018). Lung cancer staging: A concise update. Eur. Respir. J..

[23] De Leyn P., Dooms C., Kuzdzal J., Lardinois D., Passlick B., Rami-Porta R., Turna A., Schil P.V., Venuta F., Waller D. (2014). Revised ESTS guidelines for preoperative mediastinal lymph node staging for non-small-cell lung cancer. Eur. J. Cardiothorac. Surg..

[24] Vilmann P., Clementsen P., Colella S., Siemsen M., De Leyn P., Dumonceau J.-M., Herth F., Larghi A., Vasquez-Sequeiros E., Hassan C. (2015). Combined endobronchial and esophageal endosonography for the diagnosis and staging of lung cancer: European Society of Gastrointestinal Endoscopy (ESGE) Guideline, in cooperation with the European Respiratory Society (ERS) and the European Society of Thoracic Surgeons (ESTS). Endoscopy.

[25] WHO Classification of Tumours Editorial Board Thoracic Tumours. https://publications.iarc.fr/Book-And-Report-Series/Who-Classification-Of-Tumours/Thoracic-Tumours-2021.

[26] Amin M.B., Greene F.L., Edge S.B., Compton C.C., Gershenwald J.E., Brookland R.K., Meyer L., Gress D.M., Byrd D.R., Winchester D.P. (2017). The Eighth Edition AJCC Cancer Staging Manual: Continuing to build a bridge from a population-based to a more “personalized” approach to cancer staging. CA. Cancer J. Clin..

[27] Siegel R.L., Miller K.D., Wagle N.S., Jemal A. (2023). Cancer statistics, 2023. CA Cancer J. Clin..

[28] Gupta A., Omeogu C.H., Islam J.Y., Joshi A.R., Akinyemiju T.F. (2022). Association of area-level socioeconomic status and non-small cell lung cancer stage by race/ethnicity and health care-level factors: Analysis of the National Cancer Database. Cancer.

[29] Hendriks L.E., Kerr K.M., Menis J., Mok T.S., Nestle U., Passaro A., Peters S., Planchard D., Smit E.F., Solomon B.J. (2023). Oncogene-addicted metastatic non-small-cell lung cancer: ESMO Clinical Practice Guideline for diagnosis, treatment and follow-up. Ann. Oncol..

[30] Uchimura K., Yanase K., Imabayashi T., Takeyasu Y., Furuse H., Tanaka M., Matsumoto Y., Sasada S., Tsuchida T. (2021). The Impact of Core Tissues on Successful Next-Generation Sequencing Analysis of Specimens Obtained through Endobronchial Ultrasound-Guided Transbronchial Needle Aspiration. Cancers.

[31] Parente P., Carbonelli C., Biancofiore G., Sukthi A., Di Micco C.M., Vairo M., Fuso P., Taurchini M., Graziano P. (2022). Handling and standardization of EBUS needle aspiration in NSCLC patients: The value of the cell block, a monoinstitutional experience. Thorac. Cancer.

[32] Mata D.A., Harries L., Williams E.A., Hiemenz M.C., Decker B., Tse J.Y., Janovitz T., Ferguson D.C., Speece I.A., Margolis M.L. (2023). Method of Tissue Acquisition Affects Success of Comprehensive Genomic Profiling in Lung Cancer. Arch. Pathol. Lab. Med..

[33] Fernández Aceñero M.J., Díaz Del Arco C., Dinarés C., Labiano T., Tejerina E., Bernabé M.J., Forcen E., Saiz-Pardo M., Pérez P., Lozano M.D. (2023). Overview and update on molecular testing in non-small cell lung carcinoma utilizing endobronchial ultrasound-guided transbronchial needle aspiration (EBUS-TBNA) samples. Diagn. Cytopathol..

[34] Piro R., Fontana M., Casalini E., Rossi L., Simeone M.S., Ghinassi F., Ruggiero P., Pollorsi C., Taddei S., Beghe’ B. (2023). Safety and Diagnostic Accuracy of the Transnasal Approach for Endobronchial Ultrasound-Guided Transbronchial Needle Aspiration (EBUS-TBNA). Diagnostics.

[35] Muthu V., Sehgal I., Dhooria S., Prasad K., Gupta N., Aggarwal A., Agarwal R. (2019). Endobronchial ultrasound-guided transbronchial needle aspiration: Techniques and challenges. J. Cytol..

[36] Agrawal A., Ghori U., Chaddha U., Murgu S. (2022). Combined EBUS-IFB and EBUS-TBNA vs. EBUS-TBNA Alone for Intrathoracic Adenopathy: A Meta-Analysis. Ann. Thorac. Surg..

[37] Asano F., Aoe M., Ohsaki Y., Okada Y., Sasada S., Sato S., Suzuki E., Semba H., Fukuoka K., Fujino S. (2013). Complications associated with endobronchial ultrasound-guided transbronchial needle aspiration: A nationwide survey by the Japan Society for Respiratory Endoscopy. Respir. Res..

[38] Eapen G.A., Shah A.M., Lei X., Jimenez C.A., Morice R.C., Yarmus L., Filner J., Ray C., Michaud G., Greenhill S.R. (2013). Complications, Consequences, and Practice Patterns of Endobronchial Ultrasound-Guided Transbronchial Needle Aspiration: Results of the AQuIRE Registry. Chest.

[39] von Bartheld M.B., van Breda A., Annema J.T. (2014). Complication rate of endosonography (endobronchial and endoscopic ultrasound): A systematic review. Respir. Int. Rev. Thorac. Dis..

[40] Fielding D., Dalley A.J., Bashirzadeh F., Singh M., Nandakumar L., McCart Reed A.E., Black D., Kazakoff S., Pearson J.V., Nones K. (2019). Diff-Quik Cytology Smears from Endobronchial Ultrasound Transbronchial Needle Aspiration Lymph Node Specimens as a Source of DNA for Next-Generation Sequencing Instead of Cell Blocks. Respiration.

[41] Turner S.R., Buonocore D., Desmeules P., Rekhtman N., Dogan S., Lin O., Arcila M.E., Jones D.R., Huang J. (2018). Feasibility of endobronchial ultrasound transbronchial needle aspiration for massively parallel next-generation sequencing in thoracic cancer patients. Lung Cancer Amst. Neth..

[42] Wahidi M.M., Herth F., Yasufuku K., Shepherd R.W., Yarmus L., Chawla M., Lamb C., Casey K.R., Patel S., Silvestri G.A. (2016). Technical Aspects of Endobronchial Ultrasound-Guided Transbronchial Needle Aspiration: CHEST Guideline and Expert Panel Report. Chest.

[43] Righi L., Graziano P., Fornari A., Rossi G., Barbareschi M., Cavazza A., Pelosi G., Scagliotti G.V., Papotti M. (2011). Immunohistochemical subtyping of nonsmall cell lung cancer not otherwise specified in fine-needle aspiration cytology. Cancer.

[44] Hendry S., Mamotte L., Mesbah Ardakani N., Leslie C., Tesfai Y., Grieu-Iacopetta F., Izaac K., Singh S., Ardakani R., Thomas M. (2023). Adequacy of cytology and small biopsy samples obtained with rapid onsite evaluation (ROSE) for predictive biomarker testing in non-small cell lung cancer. Pathology.

[45] Xie F., Zheng X., Mao X., Zhao R., Ye J., Zhang Y., Sun J. (2019). Next-Generation Sequencing for Genotyping of Endobronchial Ultrasound-Guided Transbronchial Needle Aspiration Samples in Lung Cancer. Ann. Thorac. Surg..

[46] Zhang C., Kim R.Y., McGrath C.M., Andronov M., Haas A.R., Ma K.C., Lanfranco A.R., Hutchinson C.T., Morrissette J.J.D., DiBardino D.M. (2023). The Performance of an Extended Next Generation Sequencing Panel Using Endobronchial Ultrasound-Guided Fine Needle Aspiration Samples in Non-Squamous Non-Small Cell Lung Cancer: A Pragmatic Study. Clin. Lung Cancer.

[47] Yarmus L., Akulian J., Gilbert C., Feller-Kopman D., Lee H.J., Zarogoulidis P., Lechtzin N., Ali S.Z., Sathiyamoorthy V. (2013). Optimizing endobronchial ultrasound for molecular analysis. How many passes are needed?. Ann. Am. Thorac. Soc..

[48] Martin-Deleon R., Teixido C., Lucena C.M., Martinez D., Fontana A., Reyes R., García M., Viñolas N., Vollmer I., Sanchez M. (2021). EBUS-TBNA Cytological Samples for Comprehensive Molecular Testing in Non-Small Cell Lung Cancer. Cancers.

[49] Cho J., Choe J.G., Pahk K., Choi S., Kwon H.R., Eo J.S., Seo H.J., Kim C., Kim S. (2017). Ratio of Mediastinal Lymph Node SUV to Primary Tumor SUV in 18F-FDG PET/CT for Nodal Staging in Non-Small-Cell Lung Cancer. Nucl. Med. Mol. Imaging.

[50] Mattes M.D., Ahsanuddin S., Apte A., Moshchinsky A.B., Rizk N.P., Foster A., Wu A.J., Ashamalla H., Deasy J.O., Weber W.A. (2014). The Ratio of Lymph Node to Primary Tumor SUV on PET/CT Accurately Predicts Nodal Malignancy in Non-Small Cell Lung Cancer. Int. J. Radiat. Oncol..

[51] Stoy S.P., Segal J.P., Mueller J., Furtado L.V., Vokes E.E., Patel J.D., Murgu S. (2018). Feasibility of Endobronchial Ultrasound-guided Transbronchial Needle Aspiration Cytology Specimens for Next Generation Sequencing in Non–small-cell Lung Cancer. Clin. Lung Cancer.

[52] Pepe F., De Luca C., Smeraglio R., Pisapia P., Sgariglia R., Nacchio M., Russo M., Serra N., Rocco D., Battiloro C. (2019). Performance analysis of SiRe next-generation sequencing panel in diagnostic setting: Focus on NSCLC routine samples. J. Clin. Pathol..

[53] Roy-Chowdhuri S., Dacic S., Ghofrani M., Illei P.B., Layfield L.J., Lee C., Michael C.W., Miller R.A., Mitchell J.W., Nikolic B. (2020). Guideline From the College of American Pathologists in Collaboration with the American College of Chest Physicians, Association for Molecular Pathology, American Society of Cytopathology, American Thoracic Society, Pulmonary Pathology Society, Papanicolaou Society of Cytopathology, Society of Interventional Radiology, and Society of Thoracic Radiology. Arch. Pathol. Lab. Med..

[54] Doxtader E.E., Cheng Y.-W., Zhang Y. (2019). Molecular Testing of Non–Small Cell Lung Carcinoma Diagnosed by Endobronchial Ultrasound–Guided Transbronchial Fine-Needle Aspiration: The Cleveland Clinic Experience. Arch. Pathol. Lab. Med..

[55] Nakajima T., Yasufuku K., Fujiwara T., Yoshino I. (2016). Recent advances in endobronchial ultrasound-guided transbronchial needle aspiration. Respir. Investig..

[56] Aljohaney A., Bakhsh S., Khayat M. (2023). The Contribution of Cell Blocks in the Diagnosis of Mediastinal and Hilar Lymphadenopathy Samples From Endobronchial Ultrasound-Guided Transbronchial Needle Aspiration (EBUS-TBNA). Cureus.

[57] Sakata S., Otsubo K., Yoshida H., Ito K., Nakamura A., Teraoka S., Matsumoto N., Shiraishi Y., Haratani K., Tamiya M. (2022). Real-world data on NGS using the Oncomine DxTT for detecting genetic alterations in non-small-cell lung cancer: WJOG13019L. Cancer Sci..

[58] Froyen G., Geerdens E., Berden S., Cruys B., Maes B. (2022). Diagnostic Validation of a Comprehensive Targeted Panel for Broad Mutational and Biomarker Analysis in Solid Tumors. Cancers.

[59] Mosele F., Remon J., Mateo J., Westphalen C.B., Barlesi F., Lolkema M.P., Normanno N., Scarpa A., Robson M., Meric-Bernstam F. (2020). Recommendations for the use of next-generation sequencing (NGS) for patients with metastatic cancers: A report from the ESMO Precision Medicine Working Group. Ann. Oncol..

[60] Legras A., Barritault M., Tallet A., Fabre E., Guyard A., Rance B., Digan W., Pecuchet N., Giroux-Leprieur E., Julie C. (2018). Validity of Targeted Next-Generation Sequencing in Routine Care for Identifying Clinically Relevant Molecular Profiles in Non–Small-Cell Lung Cancer: Results of a 2-Year Experience on 1343 Samples. J. Mol. Diagn..

[61] Kage H., Kohsaka S., Shinozaki-Ushiku A., Hiraishi Y., Sato J., Nagayama K., Ushiku T., Takai D., Nakajima J., Miyagawa K. (2019). Small lung tumor biopsy samples are feasible for high quality targeted next generation sequencing. Cancer Sci..

[62] Botana-Rial M., Lojo-Rodríguez I., Leiro-Fernández V., Ramos-Hernández C., González-Montaos A., Pazos-Area L., Núñez-Delgado M., Fernández-Villar A. (2023). Is the diagnostic yield of mediastinal lymph node cryobiopsy (cryoEBUS) better for diagnosing mediastinal node involvement compared to endobronchial ultrasound-guided transbronchial needle aspiration (EBUS-TBNA)? A systematic review. Respir. Med..

[63] Yang W., Yang H., Zhang Q., Herth F.J.F., Zhang X. (2024). Comparison between Endobronchial Ultrasound-Guided Transbronchial Node Biopsy and Transbronchial Needle Aspiration: A Meta-Analysis. Respir. Int. Rev. Thorac. Dis..

[64] Kunimasa K., Matsumoto S., Nishino K., Honma K., Maeda N., Kuhara H., Tamiya M., Inoue T., Kawamura T., Kimura T. (2022). Comparison of sampling methods for next generation sequencing for patients with lung cancer. Cancer Med..

