# Supplementary material for: High-Quality Samples for Next-Generation Sequencing and PD-L1 Assessment in Non-Small Cell Lung Cancer: The Role of Endobronchial Ultrasound-Guided Transbronchial Needle Aspiration
Source: Diagnostics (Basel). 2025 Apr 22;15(9):1064. doi: 10.3390/diagnostics15091064 (PMC12071477; doi:10.3390/diagnostics15091064)
Supplement: Supplementary file 1 [file diagnostics-15-01064-s001.zip › diagnostics-3533498-supplementary.pdf]

Supplementary Table S1. Recommended metrics for DNA and RNA isolated from tumor FFPE samples.

Metric	Value
Run QC	
Key signal	65–87
Percent loading	83–92%
Raw read accurancy	98–99%
Templating QC-CF-1 Control	
Average reads per lane	16,0003–30,498
Base call accurancy	97–99%
Mean AQ20 read length (bp)	98–113
Sample QC-DNA	
MAPD	0.15–0.38
Mapped reads	150,000–1,769,932
MeanAQ20 read length (bp)	58–94
Mean read length (bp)	56–101
Uniformity of base coverage	89–99%
Sample QC-RNA	
Mapped reads	13,636–612,643
Mean read length (bp)	40–96
RNA expression controls detected	6–7
